# Long short-term memory-based forecasting of influenza epidemics using surveillance and meteorological data in Tokyo, Japan

**DOI:** 10.3389/fpubh.2025.1618508

**Published:** 2025-08-22

**Authors:** Daiki Koge, Keita Wagatsuma

**Affiliations:** ^1^Division of Bioinformatics, Department of Information Science, Graduate School of Science and Technology, Niigata University, Niigata, Japan; ^2^Institute for Research Administration, Niigata University, Niigata, Japan; ^3^Division of International Health (Public Health), Graduate School of Medical and Dental Sciences, Niigata University, Niigata, Japan

**Keywords:** influenza, meteorological factor, forecasting, epidemiology, climate change, Japan

## Abstract

**Background:**

Influenza remains a significant public health challenge worldwide, necessitating robust forecasting models to facilitate timely interventions and resource allocation. The aim of this study was to develop a long short-term memory (LSTM)-based short-term forecasting model to accurately predict weekly influenza case counts in Tokyo, Japan.

**Method:**

By using weekly time-series data on influenza incidence in Tokyo from 2000 to 2019, along with meteorological variables, we developed four distinct models to evaluate the impact of the external variables of mean temperature, relative humidity, and national public holidays. After model training, we assessed the predictive performance on an independent test dataset, using mean square error (MSE), root mean square error (RMSE), mean absolute error (MAE), and Pearson’s correlation coefficient.

**Results:**

During the study period, 1,445,944 influenza cases were analyzed. The model incorporating all three external variables demonstrated superior predictive accuracy, with an MSE of 3,646,084, RMSE of 1,909, MAE of 849, and Pearson’s correlation coefficient of 0.924. These findings underscore the substantial contribution of these external factors to improving the prediction performance.

**Conclusion:**

This study highlighted the efficacy of LSTM-based models for short-term influenza forecasting and reinforces the importance of integrating meteorological variables and national public holidays into predictive frameworks. Our optimal model provided more precise forecasts of influenza activity in Tokyo, Japan.

## 1 Introduction

Seasonal influenza remains a significant global public health challenge, contributing to annual epidemics with substantial morbidity and mortality burdens in temperate regions such as Europe and the United States (1). The infection is associated with significant systemic complications, including an increased risk of myocardial infarction, stroke, pneumonia, glycemic instability, and ischemic heart disease (2). Despite extensive research, the mechanisms driving the seasonal variability in influenza transmission are not fully understood, necessitating the development of models that incorporate genetic, environmental, and demographic factors (3, 4). Behavioral factors further shape seasonal patterns, with indoor crowding during colder months, low humidity, and temperature fluctuations hypothesized to promote outbreaks. In addition, distinct seasonal patterns in tropical regions align with rainy periods (5). These complexities underscore the need for advanced modeling techniques to better understand and predict influenza dynamics.

Traditional statistical models often fail to capture the dynamic and time-dependent nature of seasonal influenza trends. Previous studies have proposed various forecasting approaches, including statistical models (e.g., generalized linear models and autoregressive integrated moving average [ARIMA] time-series models) and mathematical models (e.g., susceptible-exposed-infected-recovered models) (6–8). By contrast, neural networks employing long short-term memory (LSTM) nodes have demonstrated substantial potential in recent years (9–12). By addressing issues such as vanishing gradients through mechanisms such as the constant error carousel and “forget gates” LSTM networks enable more robust and accurate time-series forecasting.

This study employed LSTM-based neural networks to investigate seasonal influenza epidemics in Tokyo, Japan, from 2000 to 2019. By leveraging readily available meteorological data, this study seeked to enhance the predictive accuracy and generate actionable insights for targeted public health interventions. In this study, we developed short-term forecasting models using LSTM to predict weekly influenza case counts 1 week in advance by using influenza surveillance and meteorological data from the several preceding weeks. Additionally, we report the prediction accuracy of our model for Tokyo, Japan.

## 2 Materials and methods

### 2.1 Study location

Tokyo, officially designated as the Tokyo Metropolis and serving as the capital city of Japan, is situated in the western Pacific region at a latitude of 35°N and longitude of 139°E (13). The region is characterized by a temperate climate with four distinct seasons. Summer, which extends from June to August, is typically hot and humid, whereas winter, which extends from December to February, is cold and dry. In Japan, this region primarily experiences a winter influenza epidemic. This analysis utilized weekly time-series datasets of influenza incidence and meteorological variables collected over a 20-year period (2000–2019) in Tokyo, Japan.

### 2.2 Epidemiological data

#### 2.2.1 National influenza surveillance data

We collected weekly influenza case counts in Tokyo from the Infectious Disease Weekly Report published by the Japan Institute for Health Security by the Ministry of Health, Labor, and Welfare, Tokyo, Japan (14). The reporting criteria for influenza-like illness included (1) sudden onset of symptoms, (2) fever exceeding 38.0°C, (3) upper respiratory tract inflammation, and (4) systemic symptoms. A confirmed case was identified by meeting all four criteria or at least one criterion along with a positive rapid diagnostic test.

#### 2.2.2 Meteorological data

Daily mean temperature (°C), relative humidity (%), and total rainfall (mm) were collected from the Japan Meteorological Agency single monitoring station situated in the capital city (15). These daily observations were aggregated to compute the weekly averages.

#### 2.2.3 Other data

The dataset also incorporated variables for the year, month, week, and weekly number of public holidays (Holidays).

### 2.3 Preprocessing of the dataset

#### 2.3.1 Scaling dataset

In the training process of the neural networks, the scale of the dataset values affects the stability of training convergence (16). Thus, the weekly mean number of influenza cases (Flucases) was normalized, whereas the weekly mean temperature and relative humidity (TempAve and Rh, respectively) were standardized. In particular, for the normalization of Flucases, the minimum and maximum values were fixed at 0 and 30,000, respectively, and the data were subsequently mapped to a range of 0–100.

#### 2.3.2 Data partitioning

**Figure 1:**
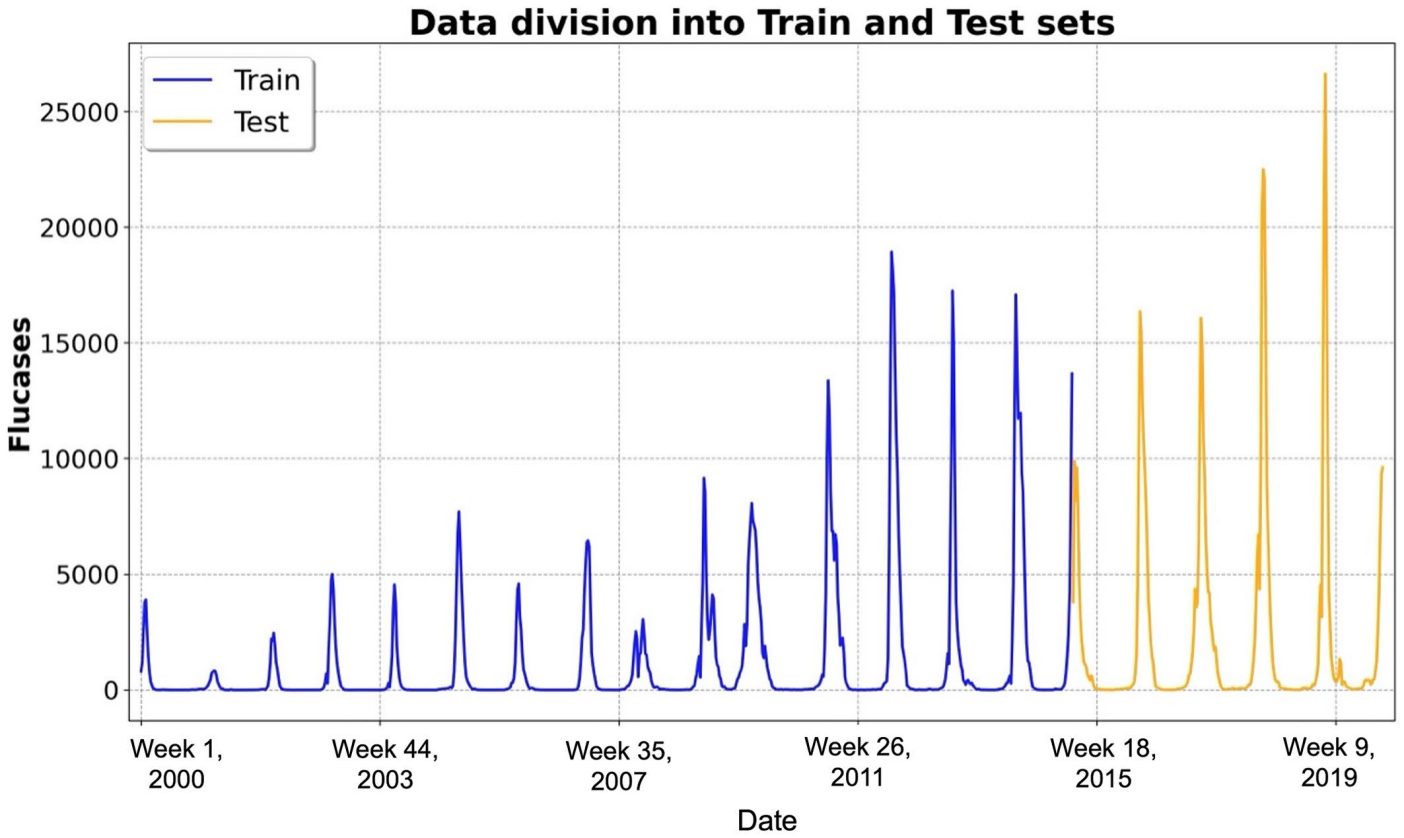
Temporal distribution of weekly influenza cases and data partitioning. This figure illustrates the temporal distribution of weekly influenza cases over the study period from 2000 to 2019, encompassing approximately 1,040 weeks (i.e., 52 weeks × 20 years). The x-axis represents the week number since 2000, whereas the y-axis denotes the number of reported influenza cases. The dataset was partitioned into two distinct subsets: the training dataset (blue line), comprising the first 740 weeks and utilized for training the long short-term memory-based forecasting models, and the test dataset (yellow line), encompassing the subsequent 260 weeks and reserved for evaluating predictive performance on unseen data. Abbreviations: Flucases, weekly mean number of influenza cases.

Our dataset, spanning 2000–2019, encompassed 1,040 weeks. The initial 740 weeks were allocated to the training set, whereas the remaining 260 weeks served as the test set for the final evaluation of the model’s predictive performance (17). Data partitioning is illustrated in Figure 1.

#### 2.3.3 Forecasting models

In this study, we introduced a series of short-term influenza forecasting models based on LSTM networks (9–12). To account for the influence of external factors—specifically, TempAve, Rh, and Holidays—on the fluctuations in Flucases, we developed four distinct models: (1) Vanilla LSTM, which utilizes only Flucases as the input and output variables; (2) Auxiliary LSTM, which extends the Vanilla LSTM by incorporating the Holidays as an auxiliary variable; (3) Vector LSTM, which employs Flucases, TempAve, and Rh as input and output variables; and (4) Auxiliary-vector (Aux-vec) LSTM:, which combines the Vector LSTM with the Holidays as an auxiliary variable.

Our LSTM models used the values of Flucases, TempAve, and Rh from up to the preceding M weeks as an input sequence, namely: $\left[y_{t-M,\;}\;y_{t-(M-1),}\cdots y_{t-2,}\;y_{t-1}\right]$ . A multilayer perceptron (MLP) was then applied to the final hidden vector $h_{t}$ of the LSTM to predict the values of Flucases, TempAve, and Rh for the subsequent week. In the Vector LSTM and Aux-vec LSTM, we defined $y\in {\mathbb{R}}^{3}$ as a vector with three values: Flucases, TempAve, and Rh. In the Vanilla LSTM and Auxiliary LSTM models, y∈ℝ denoted the scalar value of Flucases. Furthermore, in the Auxiliary LSTM and Aux-vec LSTM models, we included the Holidays for the preceding M weeks—i.e., $\;[x_{t-M,\;}\;x_{t-(M-1),}\cdots x_{t-2,}\;x_{t-1}]$ —as an auxiliary input sequence, in addition to using the number of national public holidays in the following week, $x_{t}$ , as an auxiliary input variable.

#### 2.3.4 Objective function of our models

We formulated the objective function of each model in terms of the learnable parameters w (comprising those of the LSTM and the MLP) and the observed samples. $Y≔[y_{0,}\;y_{2,}\cdots y_{T-1,}\;y_{T},]$ which denotes the sequence of observations over T weeks, where each $y_{t}$ is a vector containing influenza cases, TempAve, and Rh. Likewise, $X≔[x_{0,\;\;}\;x_{1,}\cdots x_{T-1,}\;x_{T}]$ represents the Holidays.

For the Vanilla LSTM and Vector LSTM models, we defined the log-likelihood objective function as follows:

(1) $$\log\;p(Y|w)=\log\;p(y_{0})+\sum_{t=1}^{T}\log\;p(y_{t}|,Y_{\left[t-M,t-1\right]},w),$$

where the conditional probability is assumed to follow a Gaussian distribution:

(2) $$p(y_{t}|,Y_{\left[t-M,t-1\right]},w)=N(y_{t};f(Y_{\left[t-M,t-1\right]},w),I),$$

and the Gaussian density is given by

(3) $$\begin{array}{c} \begin{array}{l} N(y_{t};f(Y_{\left[t-M,t-1\right]},w),I)\\ =\frac{1}{{\left(2\pi \right)}^{\frac{d}{2}}}\exp(-\frac{1}{2}{\left\|y_{t}-f(Y_{\left[t-M,t-1\right]},w)\right\|}^{2}), \end{array} \end{array}$$

where d=3 corresponds to the dimensionality of $y_{t}$ . In this instance, the notation $Y_{\left[t-M,t-1\right]}≔[y_{t-M,}\;y_{t-(M-1),}\cdots y_{t-2,}\;y_{t-1}]$ denotes the sequence of observations over the preceding M weeks. In our model architecture, the sequence $Y_{\left[t-M,t-1\right]}$ is provided as the input to the LSTM. The final hidden state, $h_{t}$ , produced by the LSTM is then passed to the MLP, which generates the output $f(Y_{\left[t-M,t-1\right]},w)$ that predicts $y_{t}$ .

For the Auxiliary LSTM and Aux-vec LSTM models, we extended the formulation to incorporate the auxiliary variable X (i.e., the Holidays) alongside the primary observations. In particular, the log-likelihood objective function is defined as

(4) $$\begin{array}{c} \log\;p(Y|X,w)=\log\;p(y_{0})+\sum_{t=1}^{T}\log\;p(y_{t}|Y_{\left[t-M,t-1\right]},X_{\left[t-M,t\right]},w), \end{array}$$

where the conditional distribution is assumed to be Gaussian:

(5) $$\begin{array}{c} p(y_{t}|Y_{\left[t-M,t-1\right]},w)=N(y_{t};f(Y_{\left[t-M,t-1\right]},X_{\left[t-M,t\right]},w),I), \end{array}$$

and the Gaussian density function is given by

(6) $$\begin{array}{c} \begin{array}{l} N(y_{t};f(Y_{\left[t-M,t-1\right]},w),I)\\ =\frac{1}{{\left(2\pi \right)}^{\frac{d}{2}}}\exp(-\frac{1}{2}{\left\|y_{t}-f(Y_{\left[t-M,t-1\right]},X_{\left[t-M,t\right]},w)\right\|}^{2}), \end{array} \end{array}$$

where d=3 corresponds to the dimensionality of $y_{t}$ (i.e., Flucases, TempAve, and Rh). We also defined the sequences as follows: $Y_{\left[t-M,t-1\right]}≔[y_{t-M,}\;y_{t-(M-1),}\cdots y_{t-2,}\;y_{t-1}]$ $X_{\left[t-M,t\right]}≔[x_{t-M,\;\;}\;x_{t-(M-1),}\cdots x_{t-1,}\;x_{t}]$ . In our model architecture, $Y_{\left[t-M,t-1\right]}$ and $X_{\left[t-M,t-1\right]}$ were both input into the LSTM. The final hidden state, $h_{t}$ , produced by the LSTM, in conjunction with the current auxiliary input, $x_{t}$ , is then provided to the MLP to generate the predictions $f(Y_{\left[t-M,t-1\right]},X_{\left[t-M,t-1\right]},w)$ for $y_{t}$ . We optimized parameter w of each model by using stochastic gradient descent.

#### 2.3.5 Covariate contribution assessment

To assess the relative contribution of each covariate (i.e., TempAve, Rh, and Holidays), we compared the predictive accuracy—measured by log-likelihood on the test dataset—between the full Aux-vec LSTM model and reduced models in which each covariate was systematically omitted. In addition to the four base models (Vanilla LSTM, Auxiliary LSTM, Vector LSTM, and Aux-vec LSTM), we constructed three reduced models: Model 1 (Flucases, TempAve, and Rh), Model 2 (Flucases, Rh, and Holidays), and Model 3 (Flucases, TempAve, and Holidays). Model 1 corresponds to the Vector LSTM, while Models 2 and 3 are based on the LSTM network of the Aux-vec LSTM and use the same input sequence length as the Aux-vec LSTM. Let $ℒ(M_{\mathrm{all}})$ represent the log-likelihood of the full Aux-vec LSTM model using all covariates, and ℒ(Modeli) denote the log-likelihood of the reduced Model i . The relative contribution of each covariate was quantified as the difference between $ℒ(M_{\mathrm{all}})$ and ℒ(Modeli) . We refer to this difference as the “likelihood-based contribution score” for clarity. Specifically: contribution score of Holidays $≔L(M_{\mathrm{all}})-L(\text{Model}\;1)$ ; contribution score of TempAve $≔L(M_{\mathrm{all}})-L(\text{Model}\;2)$ ; and contribution score of Rh $≔L(M_{\mathrm{all}})-L(\text{Model}\;3)$ . These scores reflect the extent to which each covariate improved model fit based on log-likelihood and should be interpreted as relative indicators of contribution, rather than absolute indicators.

#### 2.3.6 Hyperparameters

Supplementary Table 1 details the hyperparameters employed during the training of our models. The dataset was partitioned using a 75:25 split, with the initial 75% allocated for training and the remaining 25% reserved for testing (17).

#### 2.3.7 Evaluation metrics

To assess the predictive performance of our forecasting models, we employed four evaluation metrics: the mean squared error (MSE), root mean squared error (RMSE), mean absolute error (MAE), and Pearson correlation coefficient (18, 19). The variable $y_{i}$ denoted the observed values and $f_{i}$ , the corresponding model predictions for i=1,2,…,N, where N denoted the total number of observations.

The MSE is defined as the arithmetic mean of the squared differences between the observed values and their corresponding predictions. This value is formally given by

(7) $$\begin{array}{c} \mathrm{MSE}≔\frac{1}{N}\sum_{i=1}^{N}{\left(y_{i}-f_{i}\right)}^{2} \end{array}$$

where $y_{i}$ is the observed value, $f_{i}$ is the predicted value, and N is the total number of observations.

The RMSE is the square root of the MSE

(8) $$\begin{array}{c} \text{RMSE}≔\sqrt{\mathrm{MSE}}≔\sqrt{\frac{1}{N}\sum_{i=1}^{N}{\left(y_{i}-f_{i}\right)}^{2}}, \end{array}$$

thereby providing an error measure in the same units as the target variable, which facilitates a more intuitive interpretation.

MAE was calculated as the average of the absolute differences between the observed and predicted values:

(9) $$\begin{array}{c} \mathrm{MAE}≔\frac{1}{N}\sum_{i=1}^{N}{|y_{i}-f_{i}|}^{2}. \end{array}$$

Unlike the MSE, the MAE does not disproportionately emphasize larger errors and offers a more robust measure in the presence of outliers.

The Pearson’s correlation coefficient (denoted by r ) quantifies the linear relationship between the observed and predicted values. It is defined as

(10) $$\begin{array}{c} r≔\frac{\sum_{i=1}^{N}(y_{i}-\bar{y})(f_{i}-\bar{f})}{\sqrt{\sum_{i=1}^{N}{\left(y_{i}-\bar{y}\right)}^{2}}\sqrt{\sum_{i=1}^{N}{\left(f_{i}-\bar{f}\right)}^{2}}}, \end{array}$$

where $\bar{y}$ and $\bar{f}$ are the mean values of the observed and predicted datasets, respectively. This coefficient ranges from −1 to 1, with values near 1 indicating a strong positive linear correlation, values near −1 indicating a strong negative linear correlation, and values around 0 suggesting no linear correlation.

### 2.4 Software

Our experiments were conducted using Python (version 3.10.13; Python Software Foundation, Wilmington, DE, United States) in conjunction with the PyTorch deep learning framework (version 2.4.0; Linux, San Francisco, CA, United States). The source code and dataset supporting the findings of this study are publicly available at: https://github.com/daiki-ko/flu_forecast_rnn.git.

### 2.5 Ethical approval and consent to participate

This modeling study analyzed publicly available data. The datasets were de-identified and fully anonymized in advance, and the analysis of publicly available data with no identifying information did not require ethical approval.

## 3 Results

### 3.1 Descriptive statistics

Over the study period from 2000 to 2019, a total of 1,445,944 influenza cases were reported in Tokyo (Supplementary Table 2). The average number of weekly influenza cases was 1,390 (range, 0–26,635). The weekly meteorological variables spanned from 1.7°C to 31.0°C for mean temperature, from 28.7 to 94.7% for relative humidity, and from 0.0 mm to 337.5 mm for total rainfall.

### 3.2 Comparison and analysis of models

**Figure 2:**
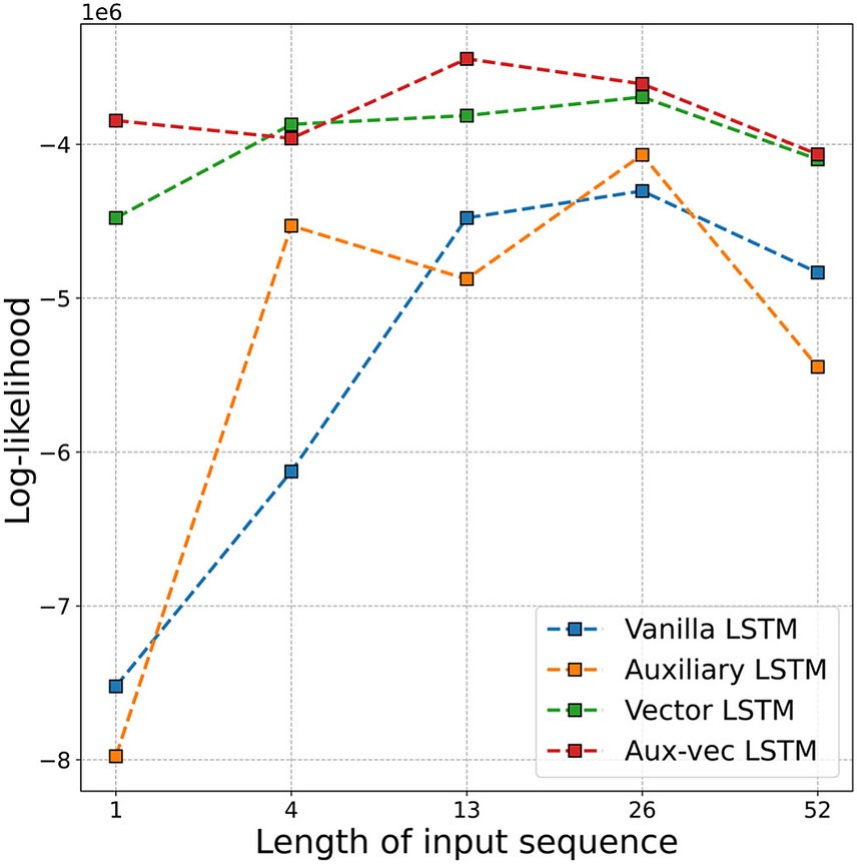
Log-likelihood values for each model are compared as a function of the input sequence length ( M ). The figure displays the log-likelihood corresponding to sequences of 1 week, 1 month (i.e., 4 weeks), 3 months (i.e., 13 weeks), 6 months (i.e., 26 weeks), and 1 year (i.e., 52 weeks). Abbreviations: LSTM, long short-term memory; Aux-vec, auxiliary-vector.

To determine the optimal input sequence length ( M ) for our LSTM models, we evaluated the log-likelihood of the prediction models for Flucases by varying M from 1 week to 52 weeks (i.e., 1 year). Figure 2 illustrates the log-likelihood values achieved in the test dataset for the different values of M . The most favorable log-likelihood values were ultimately −4,303,471 for the Vanilla LSTM at M = 26, −4,068,070 for the Auxiliary LSTM at M = 26, −3,692,631 for the Vector LSTM at M = 26, and −3,444,597 for the Aux-vec LSTM at M = 13. Considering these findings, we fixed the M value for each model at the optimal value, as indicated previously, for the subsequent analyses.

**Figure 3:**
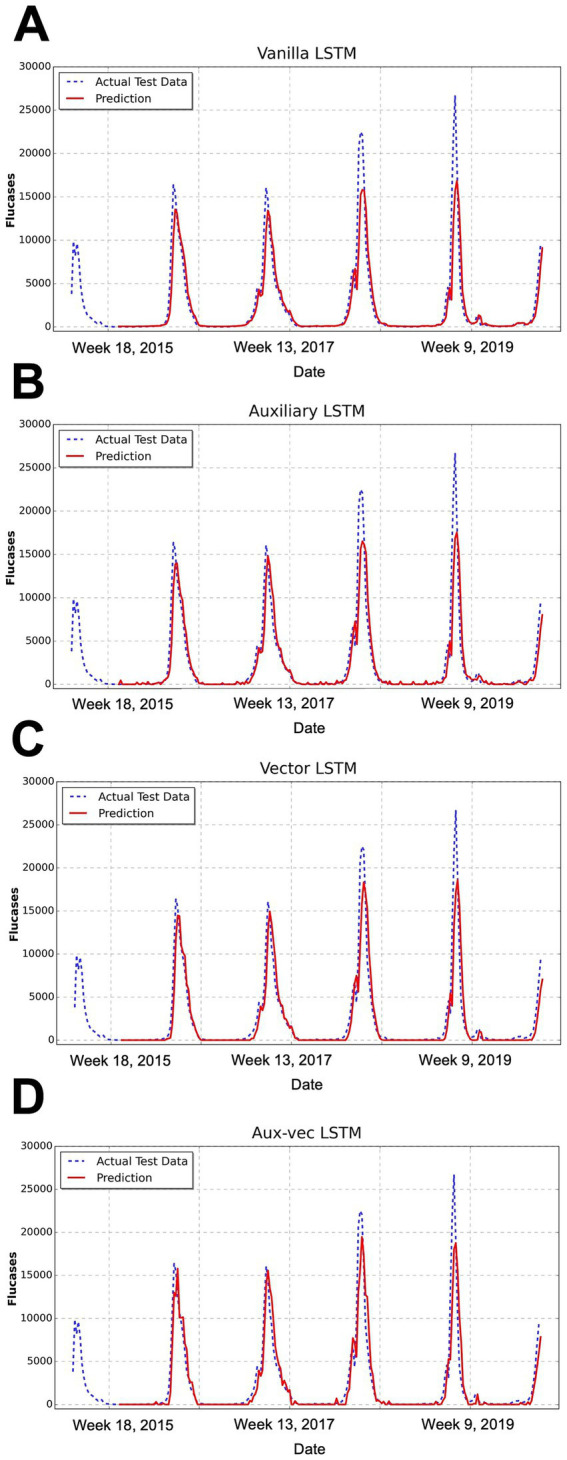
Comparison of the forecasting results of each model. This figure illustrates the temporal distribution of weekly influenza cases over the study period from 2000 to 2019, encompassing approximately 1,040 weeks (i.e., 52 weeks × 20 years). This figure presents the comparison between the predicted and actual influenza cases for the test dataset, as produced by the **(A)** Vanilla LSTM, **(B)** Auxiliary LSTM, **(C)** Vector LSTM, and **(D)** Aux-vec LSTM. The x-axis represents the week number after 2000, whereas the y-axis denotes the number of reported influenza cases. The actual test data are represented by a blue dashed line and the model’s predictions are depicted in red. Abbreviations: LSTM, long short-term memory; Aux-vec, auxiliary-vector.

We evaluated the predictive performance of the models on the test dataset by using several metrics, including MSE, RMSE, MAE, and Pearson’s correlation coefficient. The comprehensive evaluation metrics for each model are listed in Table 1, and the corresponding prediction outcomes are shown in Figure 3.

**Table 1:** Metrics for prediction for long short-term memory models.

Metric	Forecasting model			
	Vanilla LSTM	Auxiliary LSTM	Vector LSTM	Aux-vec LSTM
MSE	4,303,471	4,068,070	3,692,631	3,444,597
RMSE	2,074	2,016	1,921	1,856
MAE	880	899	886	897
Pearson’s correlation coefficient	0.916	0.916	0.924	0.927

LSTM, long short-term memory; Aux-vec, auxiliary-vector; MSE, mean squared error; RMSE, root mean squared error; MAE, mean absolute error.

The progression of the error metrics across the models revealed consistent improvement. The Vanilla LSTM model registered an MSE of 4,303,471, an RMSE of 2,074, and an MAE of 880. Incorporating the Holidays as an auxiliary variable in the Auxiliary LSTM led to modest improvements, reducing the MSE to 4,068,070, RMSE to 2,016, and MAE to 899. The performance was further enhanced in the Vector LSTM model, which integrated meteorological variables (i.e., TempAve and Rh), resulting in an MSE of 3,692,631, an RMSE of 1,921, and an MAE of 886. The most marked improvement occurred in the Aux-vec LSTM model, which combines meteorological and holiday data, achieving the lowest error metrics with an MSE of 3,444,597, an RMSE of 1,856, and an MAE of 897.

The Pearson’s correlation coefficients concomitantly exhibited a gradual increase across the models—from 0.916 for the Vanilla LSTM to 0.927 for the Aux-vec LSTM—indicating enhanced predictive accuracy. These findings illustrated that the systematic integration of external factors resulted in a steady reduction in error metrics and a corresponding improvement in the forecasting performance. In particular, the Aux-vec LSTM model demonstrated superior capability, especially in forecasting peak values, as further supported by the visual comparisons in Figure 3.

To evaluate the relative contribution of each covariate (i.e., TempAve, Rh, and Holidays), we assessed changes in predictive accuracy—quantified by log-likelihood on the test dataset—when each covariate was systematically omitted from the full Aux-vec LSTM model. As described in Section 2.3.5, we compared the performance of three reduced models (Model 1, Model 2, and Model 3), each lacking one of the covariates of interest, to the full model. The results are summarized in Supplementary Table 3. We quantified the contribution of each covariate using a “likelihood-based contribution score,” calculated as the difference in log-likelihood between the full model (incorporating all covariates) and the corresponding reduced model with a covariate omitted. Specifically, the contribution scores were as follows: TempAve, 8,341,461; Rh, 3,745,779; and Holidays, 2,480,335. These results indicate that TempAve had the greatest relative impact on model performance, followed by Rh and Holidays. The likelihood-based contribution scores are also presented graphically in Supplementary Figure 1.

In the additional analysis (Supplementary material 1), we assessed the potential contribution of weekly total rainfall by explicitly comparing forecasting metrics from models with rainfall (Supplementary Table 4) to those from our original models without rainfall (Table 1). Specifically, we compared the performance metrics (MSE, RMSE, MAE, and Pearson’s correlation coefficient) of the Vector LSTM and Aux-vec LSTM models before and after incorporating rainfall. As shown in Supplementary Table 4, adding rainfall to the Vector LSTM slightly improved the MSE from 3,692,631 (Table 1, without rainfall) to 3,679,988 (with rainfall), and similarly, RMSE marginally decreased from 1,921 to 1,918. However, MAE slightly increased from 886 to 889, and Pearson’s correlation coefficient marginally decreased from 0.924 to 0.921. For the Aux-vec LSTM, the inclusion of rainfall increased MSE from 3,444,597 (without rainfall) to 3,472,671 (with rainfall), and RMSE also slightly increased from 1,856 to 1,863. Similarly, MAE increased from 897 to 904, while Pearson’s correlation coefficient marginally decreased from 0.927 to 0.926. These comparisons indicate that although rainfall is meteorologically relevant, its addition provided minimal and inconsistent improvements in short-term influenza forecasting accuracy within this specific setting. Therefore, rainfall was retained only in the supplementary comparative analysis and was not incorporated into the main forecasting models.

## 4 Discussion

To the best of our knowledge, this study is the first systematic assessment employing LSTM-based recurrent neural networks to forecast seasonal influenza epidemics in Tokyo, Japan. In the present study, we developed four models for short-term prediction of weekly influenza case counts and rigorously evaluated their predictive performance. Each model forecasts the number of influenza cases for the subsequent week by extracting salient features from time-series data comprising influenza case counts, mean temperature, and relative humidity over the preceding weeks. Of note, the Aux-vec LSTM model, which integrated exogenous variables such as these meteorological variables and the number of national public holidays per week, exhibited superior performance, suggesting that these external factors exert a significant influence on the weekly incidence of influenza cases. To further clarify the relative contribution of each covariate, we excluded TempAve, Rh, or Holidays from the Aux-vec LSTM model and measured the increase in predictive accuracy. This covariate contribution assessment revealed that TempAve was the most influential factor, followed by Rh and Holidays.

Many studies conducted worldwide have generally supported the proposition that outdoor ambient temperature and humidity have a significant role in the transmission dynamics of influenza. For instance, a nationwide time-series analysis (20). spanning 201 Chinese cities between 2013 and 2018 quantitatively demonstrated the pronounced contribution of low temperatures to influenza incidence. In our previous large-scale epidemiological study in Japan (13), the attributable fractions of temperature and humidity were estimated to be approximately 60.0%, highlighting the substantial disease burden associated with these environmental stressors. The mechanisms by which these meteorological variables influence influenza transmission have been elucidated via several plausible pathways. First, exposure to cold weather compromises the mucociliary clearance of the nasal mucosa and promotes the ordering of lipids within the viral envelope, thereby enhancing viral stability and shedding, which then facilitates viral amplification and transmission (21). Second, extreme temperatures, whether low or high, can impair adaptive immune responses, thereby increasing host susceptibility to influenza infection (22). Third, adverse weather conditions tend to encourage individuals to remain indoors (i.e., often in air-conditioned environments), consequently elevating the frequency of close interpersonal contact and potentially augmenting transmission rates (23, 24). Although influenza is predominantly transmitted indoors, emerging evidence suggests that the short-term effects of indoor and outdoor meteorological conditions may differ (25). Therefore, future research that rigorously examines how these discrepancies influence the predictive performance of influenza transmission models is imperative. Overall, further epidemiological studies are warranted to elucidate the complex mechanisms by which ambient temperature and humidity modulate influenza transmission and to enhance the predictive accuracy of machine learning methodologies.

Numerous studies have demonstrated the utility of machine-learning-based time-series forecasting models for infectious diseases, including seasonal influenza. These investigations have primarily employed autoregressive methods [e.g., ARIMA and seasonal ARIMA (SARIMA)], hybrid deep learning models [e.g., convolutional neural network (CNN)-LSTM], and statistical approaches that integrate environmental factors. For instance, Zheng et al. (26) incorporated meteorological variables into an ARIMA with an explanatory variable (ARIMAX) model to predict the monthly influenza incidence in Fuzhou, China, and attaned an RMSE of 12.033. Chen et al. (27) similarly employed a SARIMA with exogenous factors (SARIMAX) model to forecast monthly influenza-like illness (ILI) cases in Chongqing, China, utilizing historical data in conjunction with meteorological variables (i.e., maximum and minimum temperatures and fine particulate matter). They achieved a mean absolute percentage error of 0.1903. In another study, Amendolara et al. (10) developed an LSTM-based model to predict weekly ILI rates across several major parts of the United States by using historical data and meteorological variables (i.e., mean temperature, wind speed, and precipitation), and achieved an MAE of 0.1973. Li et al. (28) applied a CNN-LSTM hybrid model to forecast weekly ILI rates in Hebei Province, China, relying solely on historical ILI data. They reported an MAE of 0.4388. Our study extended this body of research by integrating meteorological variables and national public holiday data into an LSTM-based model to enhance the accuracy of weekly influenza forecasts for Tokyo, Japan. Shi et al. (29) noted that holiday travel may trigger secondary epidemic peaks through increased interpersonal contact.

In our supplementary comparative analysis, we evaluated the contribution of weekly total rainfall to influenza forecasting accuracy by comparing models with and without rainfall. Incorporating rainfall into our forecasting models yielded minimal and inconsistent changes in performance. This limited predictive contribution of rainfall observed in Tokyo, an urban area with a temperate climate, may be partly explained by previous evidence showing climate-dependent responses of influenza activity. For instance, studies indicate that temperate regions generally have stronger associations between influenza incidence and temperature or humidity compared to subtropical regions (30). This could reflect lower average temperatures and humidity levels in temperate climates, making these meteorological factors more influential than rainfall. Nonetheless, previous studies conducted in tropical and subtropical regions, such as Singapore and Hong Kong, have reported stronger associations between rainfall and influenza activity (31, 32). According to the previous study, the role of rainfall in influenza transmission may not be through direct effects on virus survivorship or host susceptibility; rather, rainfall-induced changes in social behaviors—such as increased indoor activities on rainy days—may facilitate influenza transmission through greater interpersonal contact (32). While rainfall is meteorologically relevant, its effect in short-term influenza forecasting, at least within Tokyo’s urban temperate context, seems comparatively limited. Further investigations using alternative rainfall metrics or studies conducted in other climatic zones would be beneficial to more precisely evaluate subtle influences of rainfall on influenza transmission dynamics.

This study had some limitations. First, it did not account for multiple nonmeteorological variables influencing influenza transmission dynamics such as human behavior, travel patterns, population immunity, virus subtypes, viral variability, and public health and social interventions (33). However, modeling these factors at the national or prefectural scale for an early warning system in Japan is challenging. Second, the analysis relied solely on data from Tokyo, necessitating validation by using datasets from multiple regions. Third, the meteorological data were collected from fixed weather monitoring stations, which may have introduced an exposure measurement bias and potentially reduced the precision and statistical power of our findings. Forth, this study was constrained by a noticeable discrepancy in the distribution of influenza case counts between the training and testing datasets, which may have contributed to reduced predictive accuracy during periods of unusually high incidence in the later stages of the time series. Addressing this distributional mismatch—potentially through the use of expanded or rebalanced training datasets—may improve model performance in future study. Finally, as in all ecological studies, our results are inherently susceptible to ecological fallacies.

## 5 Conclusion

We developed a series of LSTM-based short-term influenza forecasting models for Tokyo, Japan. Our four models demonstrated robust performances in predicting influenza cases over short periods. Of note, the Aux-vec LSTM model, which integrated meteorological variables and the weekly number of public holidays, exhibited the best predictive performance. The evaluation results underscore that external factors such as temperature, relative humidity, and the number of days off per week are crucial for accurate forecasting. These findings enhance the precision of influenza predictions in Tokyo, and highlight the potential of the Aux-vec LSTM model for epidemic forecasting of viruses influenced by these external factors. Moreover, our covariate contribution assessment further substantiated that mean temperature had the most significant impact on prediction accuracy, followed by relative humidity and national public holidays. This highlights the robustness of incorporating temperature-related features into influenza forecasting models, particularly in temperate regions like Tokyo. More precise influenza predictions could be useful in planning targeted vaccination campaigns, in healthcare resource planning, and in enhancing public health messaging and preventive measures.

## Data Availability

The raw data supporting the conclusions of this article will be made available by the authors, without undue reservation.

## References

[1] Lafond KE Porter RM Whaley MJ Suizan Z Ran Z Aleem MA. Global burden of influenza-associated lower respiratory tract infections and hospitalizations among adults: a systematic review and meta-analysis. PLoS Med. (2021) 18:e1003550. doi: 10.1371/JOURNAL.PMED.1003550, PMID: 33647033 PMC7959367

[2] Macias AE McElhaney JE Chaves SS Nealon J Nunes MC Samson SI. The disease burden of influenza beyond respiratory illness. Vaccine. (2021) 39:A6–A14. doi: 10.1016/j.vaccine.2020.09.048 33041103 PMC7545338

[3] Tamerius J Nelson MI Zhou SZ Viboud C Miller MA Alonso WJ. Global influenza seasonality: reconciling patterns across temperate and tropical regions. Environ Health Perspect. (2011) 119:439–45. doi: 10.1289/ehp.1002383, PMID: 21097384 PMC3080923

[4] Lofgren E Fefferman NH Naumov YN Gorski J Naumova EN. Influenza seasonality: underlying causes and modeling theories. J Virol. (2007) 81:5429–36. doi: 10.1128/jvi.01680-06, PMID: 17182688 PMC1900246

[5] Yuan H Kramer SC Lau EHY Cowling BJ Yang W. Modeling influenza seasonality in the tropics and subtropics. PLoS Comput Biol. (2021) 17:e1009050. doi: 10.1371/journal.pcbi.1009050, PMID: 34106917 PMC8216520

[6] Xu Q Gel YR Ramirez LLR Nezafati K Zhang Q Tsui KL. Forecasting influenza in Hong Kong with Google search queries and statistical model fusion. PLoS One. (2017) 12:e0176690. doi: 10.1371/journal.pone.0176690 28464015 PMC5413039

[7] He Z Tao H. Epidemiology and ARIMA model of positive-rate of influenza viruses among children in Wuhan, China: a nine-year retrospective study. Int J Infect Dis. (2018) 74:61–70. doi: 10.1016/j.ijid.2018.07.003, PMID: 29990540

[8] Saito MM Imoto S Yamaguchi R Sato H Nakada H Kami M. Extension and verification of the SEIR model on the 2009 influenza a (H1N1) pandemic in Japan. Math Biosci. (2013) 246:47–54. doi: 10.1016/j.mbs.2013.08.009, PMID: 24012502

[9] Yu Y Si X Hu C Zhang J. A review of recurrent neural networks: Lstm cells and network architectures. Neural Comput. (2019) 31:1235–70. doi: 10.1162/neco_a_01199, PMID: 31113301

[10] Amendolara AB Sant D Rotstein HG Fortune E. LSTM-based recurrent neural network provides effective short term flu forecasting. BMC Public Health. (2023) 23:1788. doi: 10.1186/s12889-023-16720-6, PMID: 37710241 PMC10500783

[11] Kara A. Multi-step influenza outbreak forecasting using deep LSTM network and genetic algorithm. Expert Syst Appl. (2021) 180:115153. doi: 10.1016/j.eswa.2021.115153, PMID: 40782729

[12] Zhu H Chen S Lu W Chen K Feng Y Xie Z. Study on the influence of meteorological factors on influenza in different regions and predictions based on an LSTM algorithm. BMC Public Health. (2022) 22:2335. doi: 10.1186/s12889-022-14299-y, PMID: 36514013 PMC9745690

[13] Wagatsuma K Madaniyazi L Sheng Ng CF Saito R Hashizume M. Characterizing the seasonal influenza disease burden attributable to climate variability: a nationwide time-series modelling study in Japan, 2000–2019. Environ Res. (2024) 263:120065. doi: 10.1016/j.envres.2024.120065, PMID: 39341540

[14] National Institute of Infectious Diseases. *National Epidemiological Surveillance of Infectious Diseases*. (2024). Available online at: https://www.niid.go.jp/niid/ja/idwr.html (Accessed April 20, 2024].

[15] Japan Meteorological Agency. *Meteorological Data Search*. (2024). Available online at: https://www.jma.go.jp/jma/index.html (Accessed April 20, 2024).

[16] Allen-Zhu Z Li Y Song Z. On the convergence rate of training recurrent neural networks. Adv Neural Inf Process Syst. (2019) 32:12065. doi: 10.48550/arXiv.1810.12065

[17] Korjus K Hebart MN Vicente R. An efficient data partitioning to improve classification performance while keeping parameters interpretable. PLoS One. (2016) 11:e0161788. doi: 10.1371/journal.pone.0161788, PMID: 27564393 PMC5001642

[18] Karunasingha DSK. Root mean square error or mean absolute error? Use their ratio as well. Inf Sci (NY). (2022) 585:609–29. doi: 10.1016/j.ins.2021.11.036

[19] Sedgwick P. Pearson’s correlation coefficient. BMJ. (2012) 345:345. doi: 10.1136/bmj.e4483, PMID: 40771509

[20] Yin Y Lai M Lu K Jiang X Chen Z Li T. Association between ambient temperature and influenza prevalence: a nationwide time-series analysis in 201 Chinese cities from 2013 to 2018. Environ Int. (2024) 189:108783. doi: 10.1016/j.envint.2024.108783, PMID: 38823156

[21] Eccles R. An explanation for the seasonality of acute upper respiratory tract viral infections. Acta Otolaryngol. (2002) 122:183–91. doi: 10.1080/00016480252814207, PMID: 11936911

[22] Kreijtz JHCM Fouchier RAM Rimmelzwaan GF. Immune responses to influenza virus infection. Virus Res. (2011) 162:19–30. doi: 10.1016/j.virusres.2011.09.022, PMID: 21963677

[23] Fuhrmann C. The effects of weather and climate on the seasonality of influenza: what we know and what we need to know. Geogr Compass. (2010) 4:718–30. doi: 10.1111/j.1749-8198.2010.00343.x

[24] Willem L van Kerckhove K Chao DL Hens N Beutels P. A nice day for an infection? Weather conditions and social contact patterns relevant to influenza transmission. PLoS One. (2012) 7:e48695. doi: 10.1371/journal.pone.0048695, PMID: 23155399 PMC3498265

[25] Lei H Yang M Dong Z Hu K Chen T Yang L. Indoor relative humidity shapes influenza seasonality in temperate and subtropical climates in China. Int J Infect Dis. (2023) 126:54–63. doi: 10.1016/j.ijid.2022.11.023, PMID: 36427703

[26] Zheng X Chen Q Sun M Zhou Q Shi H Zhang X. Exploring the influence of environmental indicators and forecasting influenza incidence using ARIMAX models. Front Public Health. (2024) 12:1441240. doi: 10.3389/fpubh.2024.1441240, PMID: 39377003 PMC11456462

[27] Chen H Xiao M. Seasonality of influenza-like illness and short-term forecasting model in Chongqing from 2010 to 2022. BMC Infect Dis. (2024) 24:432. doi: 10.1186/s12879-024-09301-4, PMID: 38654199 PMC11036656

[28] Li G Li Y Han G Jiang C Geng M Guo N. Forecasting and analyzing influenza activity in Hebei Province, China, using a CNN-LSTM hybrid model. BMC Public Health. (2024) 24:2171. doi: 10.1186/s12889-024-19590-8, PMID: 39135162 PMC11318307

[29] Shi P Keskinocak P Swann JL Lee BY. The impact of mass gatherings and holiday traveling on the course of an influenza pandemic: a computational model. BMC Public Health. (2010) 10:778. doi: 10.1186/1471-2458-10-778, PMID: 21176155 PMC3022852

[30] Wang XL Yang L He DH Chiu AP Chan KH Chan KP. Different responses of influenza epidemic to weather factors among Shanghai, Hong Kong, and British Columbia. Int J Biometeorol. (2017) 61:1043–53. doi: 10.1007/s00484-016-1284-y, PMID: 28180957

[31] Chew FT Doraisingham S Ling AE Kumarasinghe G Lee BW. Seasonal trends of viral respiratory tract infections in the tropics. Epidemiol Infect. (1998) 121:121–8. doi: 10.1017/S0950268898008905, PMID: 9747763 PMC2809482

[32] Soebiyanto RP Adimi F Kiang RK. Modeling and predicting seasonal influenza transmission in warm regions using climatological parameters. PLoS One. (2010) 5:e9450. doi: 10.1371/journal.pone.0009450, PMID: 20209164 PMC2830480

[33] Bonacina F Boëlle PY Colizza V Lopez O Thomas M Poletto C. Global patterns and drivers of influenza decline during the COVID-19 pandemic. Int J Infect Dis. (2023) 128:132–9. doi: 10.1016/j.ijid.2022.12.042, PMID: 36608787 PMC9809002

