# Supplementary material for: Long short-term memory-based forecasting of influenza epidemics using surveillance and meteorological data in Tokyo, Japan
Source: Front Public Health. 2025 Aug 22;13:1618508. doi: 10.3389/fpubh.2025.1618508 (PMC12411156; doi:10.3389/fpubh.2025.1618508)
Supplement: Supplementary file 1 [file Data_Sheet_1.docx]

Supplementary Material

**Supplementary Materials**

## Supplementary Material 1. Comparison model

To further assess the potential contribution of additional meteorological variables, we conducted a comparative analysis by incorporating weekly total rainfall into the input features of each long short-term memory (LSTM) model. This supplementary section aims to evaluate model performance with and without rainfall as a covariate. Specifically, we reassessed model performance across four configurations: (1) Vanilla LSTM (baseline), (2) Auxiliary LSTM incorporating the weekly number of public holidays (Holidays), (3) Vector LSTM incorporating weekly mean temperature (TempAve), weekly mean relative humidity (Rh), and total rainfall, and (4) auxiliary-vector (Aux-vec) LSTM combining the Vector LSTM inputs with national public holidays.

## Supplementary Tables

**Supplementary Table 1. Hyperparameters employed during the training of our models.**

| Hyperparameter | Choice |
| --- | --- |
| Batch size | 50 |
| Optimizer | Adam |
| Learning rate | 0.001 |
| Epochs | 500 |
| Training samples for parameter optimization | 632 samples |
| Validation samples for early stopping | 148 samples |
| Test samples for evaluation | 260 samples |

Abbreviations: Adam, adaptive moment estimation

**Supplementary Table 2. Descriptive statistics for the weekly number of confirmed influenza cases (n = 1,445,944) and meteorological variables from 2000 to 2019 in Tokyo, Japan.**

| Variable | Mean | SD | Min | Max |
| --- | --- | --- | --- | --- |
| Weekly number of confirmed influenza cases | 1,390 | 3,279 | 0 | 26,635 |
| Weekly mean temperature (°C) | 16.6 | 7.6 | 1.7 | 31.0 |
| Weekly mean relative humidity (%) | 62.1 | 12.2 | 28.7 | 94.7 |
| Weekly mean total rainfall (mm) | 31.4 | 41.3 | 0.0 | 337.5 |

Abbreviations: SD, standard deviation; Min, minimum; Max, maximum

**Supplementary Table 3. Metrics for long short-term memory models for covariate importance analysis.**

| Metric | Forecasting model | | |
| --- | --- | --- | --- |
|  | Model 1: Flucases + TempAve + Rh  (without Holidays) | Model 2: Flucases + Rh + Holidays  (without TempAve) | Model 3: Flucases + TempAve + Holidays  (without Rh) |
| MSE | 3,692,631 | 4,278,744 | 3,819,175 |
| RMSE | 1,921 | 2,069 | 1,954 |
| MAE | 886 | 963 | 925 |
| Pearson’s correlation coefficient | 0.924 | 0.915 | 0.929 |

Abbreviations: LSTM, long short-term memory; Aux‐vec, auxiliary-vector; MSE, mean squared error; RMSE, root mean squared error; MAE, mean absolute error; Holidays, weekly number of public holidays; Rh, Weekly mean relative humidity; TempAve, Weekly mean temperature

**Supplementary Table 4. Metrics for prediction for long short-term memory models.**

| Metric | Forecasting model | | | | |
| --- | --- | --- | --- | --- | --- |
|  | Vanilla LSTM | Auxiliary LSTM | Vector LSTM | | Aux-vec LSTM |
| MSE | 4,303,471 | 4,068,070 | | 3,679,988 | 3,472,671 |
| RMSE | 2,074 | 2,016 | | 1,918 | 1,863 |
| MAE | 880 | 899 | | 889 | 904 |
| Pearson’s correlation coefficient | 0.916 | 0.916 | | 0.921 | 0.926 |

Abbreviations: LSTM, long short-term memory; Aux‐vec, auxiliary-vector; MSE, mean squared error; RMSE, root mean squared error; MAE, mean absolute error

## Supplementary Figures

**Supplementary Figure 1. Contribution score of each covariate.**


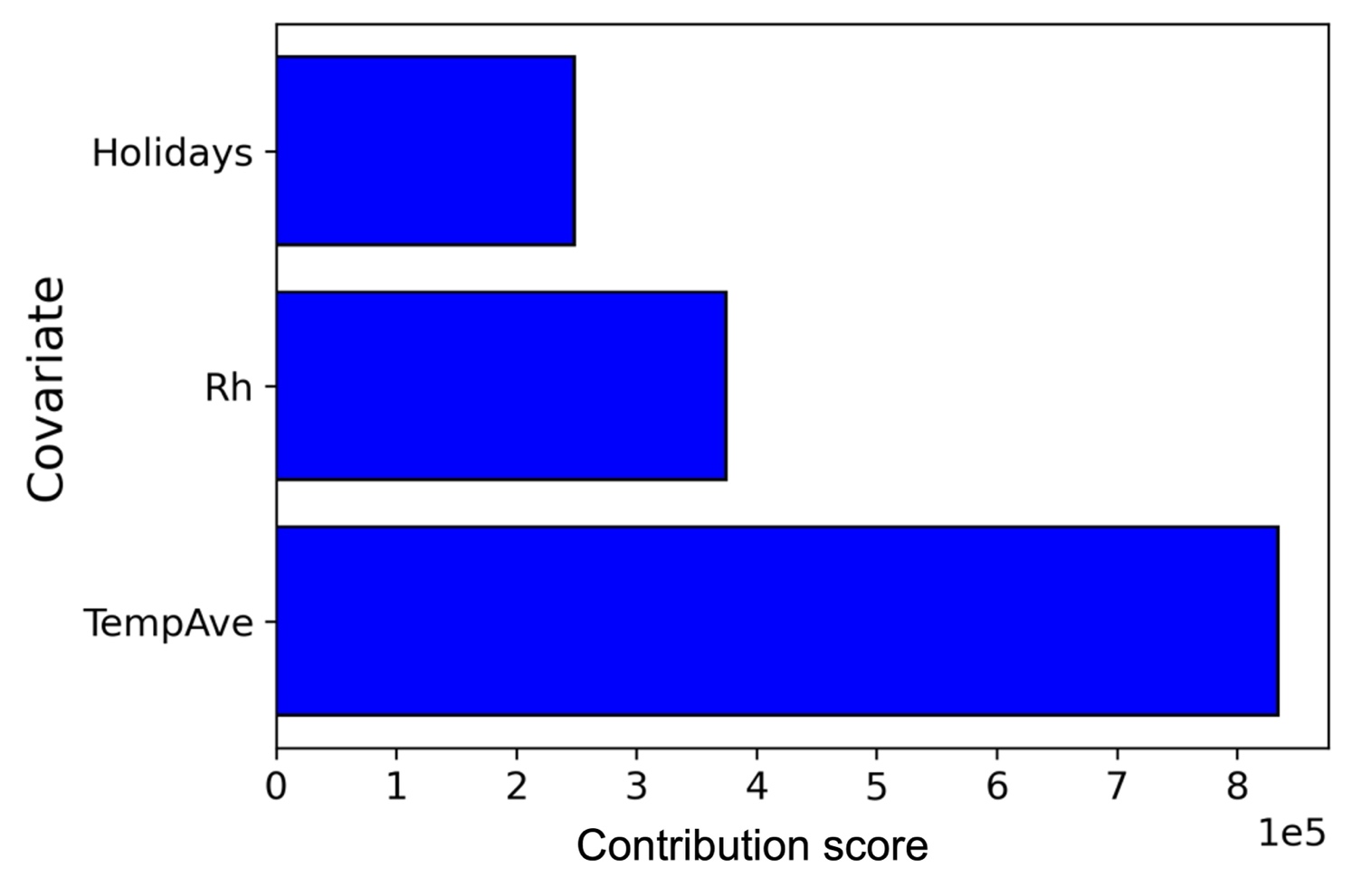


Abbreviations: Holidays, weekly number of public holidays; Rh, Weekly mean relative humidity; TempAve, Weekly mean temperature
